# Hemopexin is required for adult neurogenesis in the subventricular zone/olfactory bulb pathway

**DOI:** 10.1038/s41419-018-0328-0

**Published:** 2018-02-15

**Authors:** Yanling Zhu, Yang Qiu, Mengjia Chen, Yi Zhang, Li Cao, Zhida Su, Yimin Yuan, Aijun Huang, Yinyan Pu, Cheng He

**Affiliations:** Institute of Neuroscience, Key Laboratory of Molecular Neurobiology of Ministry of Education and the Collaborative Innovation Center for Brain Science, Second Military Medical University, 200433 Shanghai, China

## Abstract

The neural stem cells (NSCs) of the subventricular zone (SVZ) reside within a specialized niche critical for neurogenesis. Hemopexin, a plasma glycoprotein, has been extensively studied as a heme scavenger at the systemic level. However, little is known about its function in the central nervous system, especially in neurogenesis. In the present study, we demonstrate that deletion of hemopexin leads to neurogenic abnormalities in the SVZ/olfactory bulb (OB) pathway. The lateral ventricle is enlarged in hemopexin-deficient mice, and more apoptosis was observed in Dcx+ cells. Lineage differentiation of NSCs was also inhibited in the SVZ of hemopexin-deficient mice, with more stem cells stayed in an undifferentiated, GFAP+ radial glia-like cell stage. Moreover, hemopexin deletion resulted in impaired neuroblast migration in the rostral migratory stream. Furthermore, exogenous hemopexin protein inhibited apoptosis and promoted the migration and differentiation of cultured NSCs. Finally, immunohistochemical analysis demonstrated that deletion of hemopexin reduced the number of interneurons in the OB. Together, these results suggest a new molecular mechanism for the NSC niche that regulates adult neurogenesis in the SVZ/OB pathway. Our findings may benefit the understanding for olfactory system development.

## Introduction

The subventricular zone (SVZ) of the lateral ventricle is one of the most important regions for neurogenesis in the adult mammalian brain^1,2^. Neural stem cells (NSCs)/progenitors in the SVZ are classified as three major types with lineage-relationship: type B, C, and A cells^3^. Type B cells are stem cells that possess the ultrastructural characteristics of brain astrocytes and express glial fibrillary acidic protein (GFAP)^4,5^. The slowly dividing type B cells produce transit-amplifying type C cells, which are MASH1 positive and divide rapidly^3^. Type C cells, in turn, give rise to type A cells, a population of doublecortin (Dcx)-positive neuroblasts^4^. The neuroblasts migrate anteriorly in chains along the rostral migratory stream (RMS) to the olfactory bulb (OB), where they differentiate into different subtypes of local interneurons, such as GAD67+ GABAergic granule neurons, GABAergic periglomerular neurons, and TH+ dopaminergic periglomerular neurons, which integrate into OB circuits^6,7^.

The NSC niche is a complex microenvironment that is organized to favor specific cell–cell interactions and access to the cerebral microvasculature, extracellular matrix components, meninges, and cerebrospinal fluid (CSF). Recently, the three-dimensional structure of the adult stem cell niche in the SVZ has begun to be defined^8^. The SVZ has been found to be confined by a layer of ciliated ependymal cells on its ventricular side and an extensive planar network of blood vessels (called the SVZ plexus) on its parenchymal side^9–11^. NSCs in the SVZ reside in the center of a pinwheel structure that is surrounded by ependymal cells that maintain NSC properties by producing soluble factors^12^. The apical endfeet of NSCs contact CSF directly, thereby sensing environmental cues contained in CSF^13,14^. In addition to ependymal cells and CSF, NSCs in the SVZ extend processes in direct contact with blood vessels, which occurs frequently in areas that lack astrocyte and pericyte coverage. And along the RMS, blood vessels in the dorsal SVZ can also serve as a migratory scaffold to guide neuroblast chains as they migrate^15–17^. This enables NSCs to respond to factors in the circulation^3,11^. Many factors produced by the choroid plexus are reported to affect NSCs in the SVZ^18–20^. Despite many fundamentally important mechanism have been discovered, the process by which the niche regulates neurogenic needs further study.

Hemopexin (Hpx) is a plasma glycoprotein produced mainly by the liver that is widely known as a heme scavenger at the systemic level^21,22^. Hpx is also primarily expressed by ependymal cells that line the ventricular system and by hippocampal neurons^23^, which located near the two major neurogenic regions in the adult mammalian brain. And in a proteomic study, the Hpx protein was identified in human CSF^24^. However, little is known about the functions of Hpx in the central nervous system (CNS)^25,26^. Considering its ependymal expression, its presence in CSF, and its high concentration in plasma, all of which are components of the NSC niche in the SVZ, we hypothesized that Hpx may perform important functions during neurogenesis.

In the present study, we demonstrate that Hpx is required for the survival, migration, and differentiation of neural precursors in the SVZ/OB pathway, which suggests that a novel molecular mechanism underlies adult stem cell niche regulation of neurogenesis.

## Results

### Hemopexin deletion influences the stem pool in the anterior SVZ

**Fig. 1:**
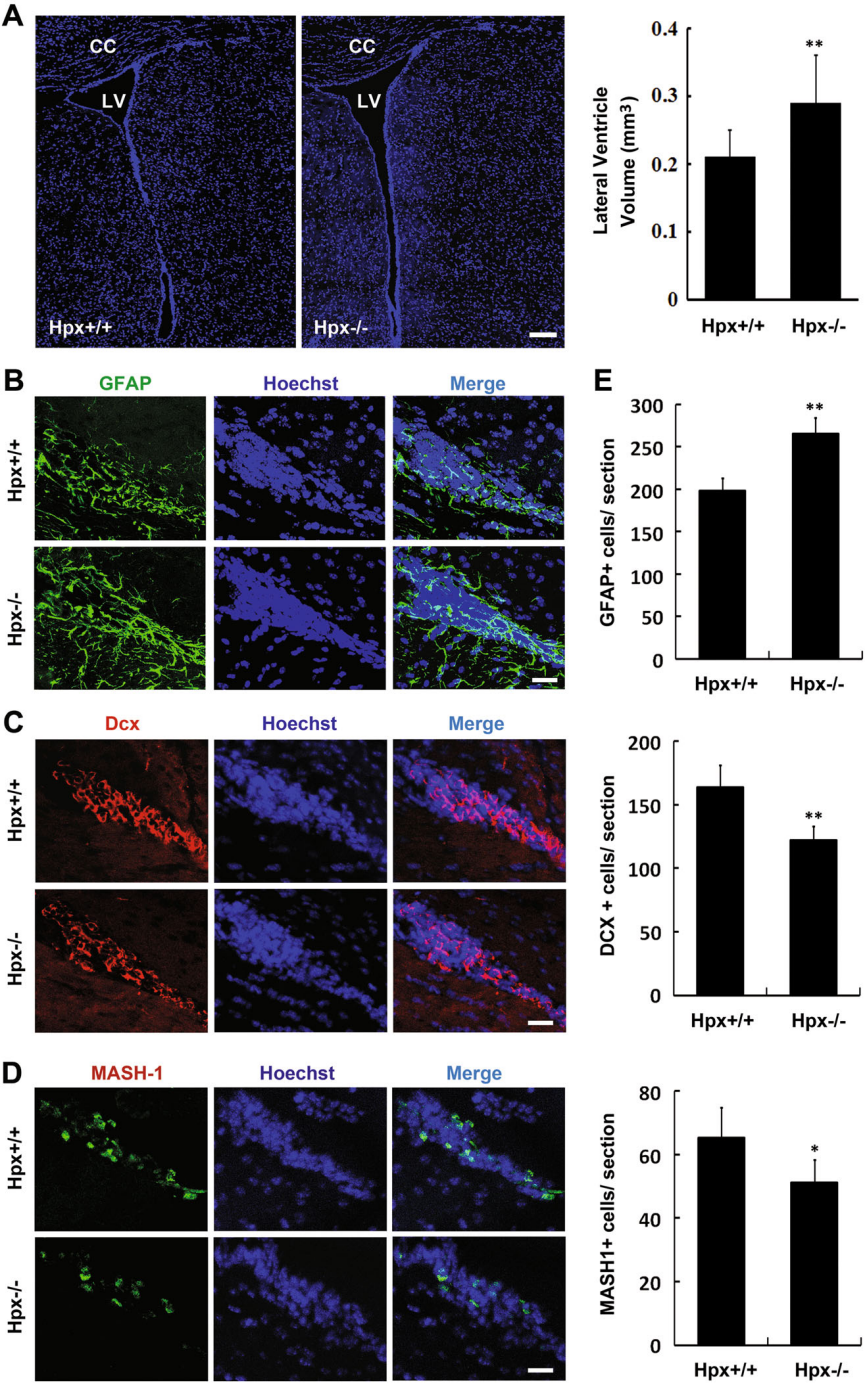
Abnormality of the stem cell pool in the SVZa in Hpx-deficient (Hpx^−/−^) mice. **a** Representative images of Hoechst-stained coronal sections from brains of wild-type (Hpx^+/+^) and Hpx^−/−^ mice were used to visualize the size of the lateral ventricles. Stereological quantification of the volume of the lateral ventricles was performed. Hpx^−/−^ brains showed enlarged lateral ventricles. *n* = 9. **b**–**d** The lineage differentiation of NSCs/progenitors was inhibited, and more stem cells remained in an undifferentiated GFAP+ stage. Anti-GFAP (**b**, green), anti-Dcx (**c**, red), and anti-MASH1 (**d**, red) were used to label stem cells/progenitors at different stages in the SVZ in coronal sections of brains. **e** The number of GFAP+ B cells, MASH1+ C cells, and Dcx+ A cells were measured. GFAP+ stem cells were increased, while Dcx+ and MASH1+ cells were decreased in Hpx^−/−^ mice. *n* = 5. *p < 0.05, **p < 0.01. Scale bar: 100 μm in **a**; 20 μm in **b**–**d**. LV lateral ventricle, CC corpus callosum

The SVZ of the lateral ventricle is one of the most important regions for adult neurogenesis in the mammalian brain. As reported, abnormal changes of the stem cells in the SVZ may influence the size of the lateral ventricles^27,28^. Accordingly, we first measured the volume of the lateral ventricles. As shown in Fig. 1a, the brains of Hpx-deleted mice (Hpx^−/−^) had enlarged lateral ventricles compared to Hpx^+/+^ mice.

The SVZ lineage is derived from slowly dividing type B cells, which produce the fast-proliferating type C cells that in turn give rise to type A neuroblasts^3^. To further examine the stem cell pool in Hpx-deficient mice, we performed immunohistochemical analysis. We used anti-GFAP to label radial glia-like cells (Fig. 1b), anti-doublecortin to label neuroblasts (Fig. 1c), and anti-MASH1 to label transit-amplifying cells (Fig. 1d). Quantification of the number of each cell type in the SVZ showed that the number of GFAP-positive B cells increased, while the numbers of MASH1-positive C cells and Dcx-positive A cells both decreased in Hpx^−/−^ mice compared to wild-type mice (Fig. 1e).

Taken together, these data indicate that the stem pool in the anterior SVZ (SVZa) became abnormal after Hpx deletion. In particular, the lineage differentiation of stem cells/progenitors was affected in the SVZ, with more stem cells staying in the undifferentiated GFAP+ radial glia-like cell stage and fewer cells becoming MASH1+ transit-amplifying cells or Dcx+ neuroblasts.

### Hemopexin deletion increases the apoptosis of Dcx+ cells

**Fig. 2:**
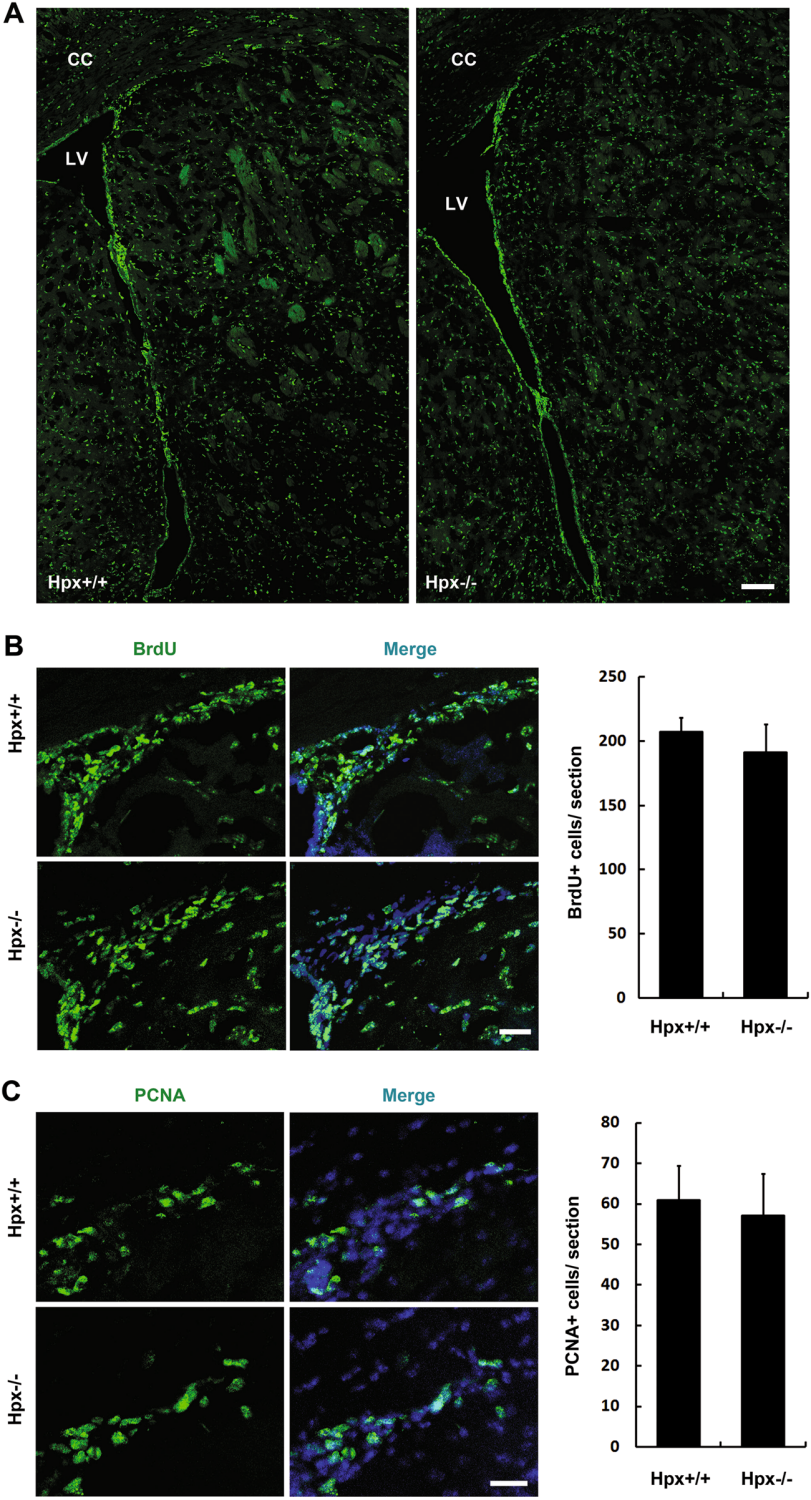
Proliferation of stem cells/progenitors in SVZa was not changed by hemopexin deletion. **a** Mice were dosed with BrdU and killed 2 h later. Every fifth coronal section from each brain was labeled with anti-BrdU (green) to analyze proliferation. **b** High-magnification images showing the BrdU-positive cells in the SVZa. Hoechst staining (blue) was used to identify nuclei. Quantification of BrdU+ cells in the SVZa (right). No significant difference was found between Hpx^+/+^ and Hpx^−/−^ mice. *n* = 4. **c** High-magnification images showing the PCNA-positive cells in the SVZa. Hoechst staining (blue) was used to identify nuclei. Quantification of PCNA+ cells in the SVZa (right). No significant difference was found between Hpx^+/+^ and Hpx^−/−^ mice. *n* = 3. Scale bar: 100 μm in **a**; 20 μm in **b**, **c**. LV lateral ventricle, CC corpus callosum

To gain insight into the abnormalities observed in the stem pool in the SVZa of Hpx-deficient mice, we examined proliferation and apoptosis of NSCs/progenitors. Proliferation was assessed by 2 h of BrdU treatment, which labels cells in the S phase of the cell cycle, and then BrdU-positive cells in the SVZa were counted. As shown in Fig. 2a, b, no significant difference was observed in the number of BrdU-positive cells between Hpx^+/+^ and Hpx^−/−^ mice. To further confirm this, we also used PCNA antibody to label the proliferation cells. No statistical difference was found in the number of the PCNA-positive cells in the SVZa between Hpx +/+ and Hpx^−/−^ mice (Fig. 2c). These data suggesting that Hpx deletion does not affect the proliferation of NSCs/progenitors in the SVZa.

**Fig. 3:**
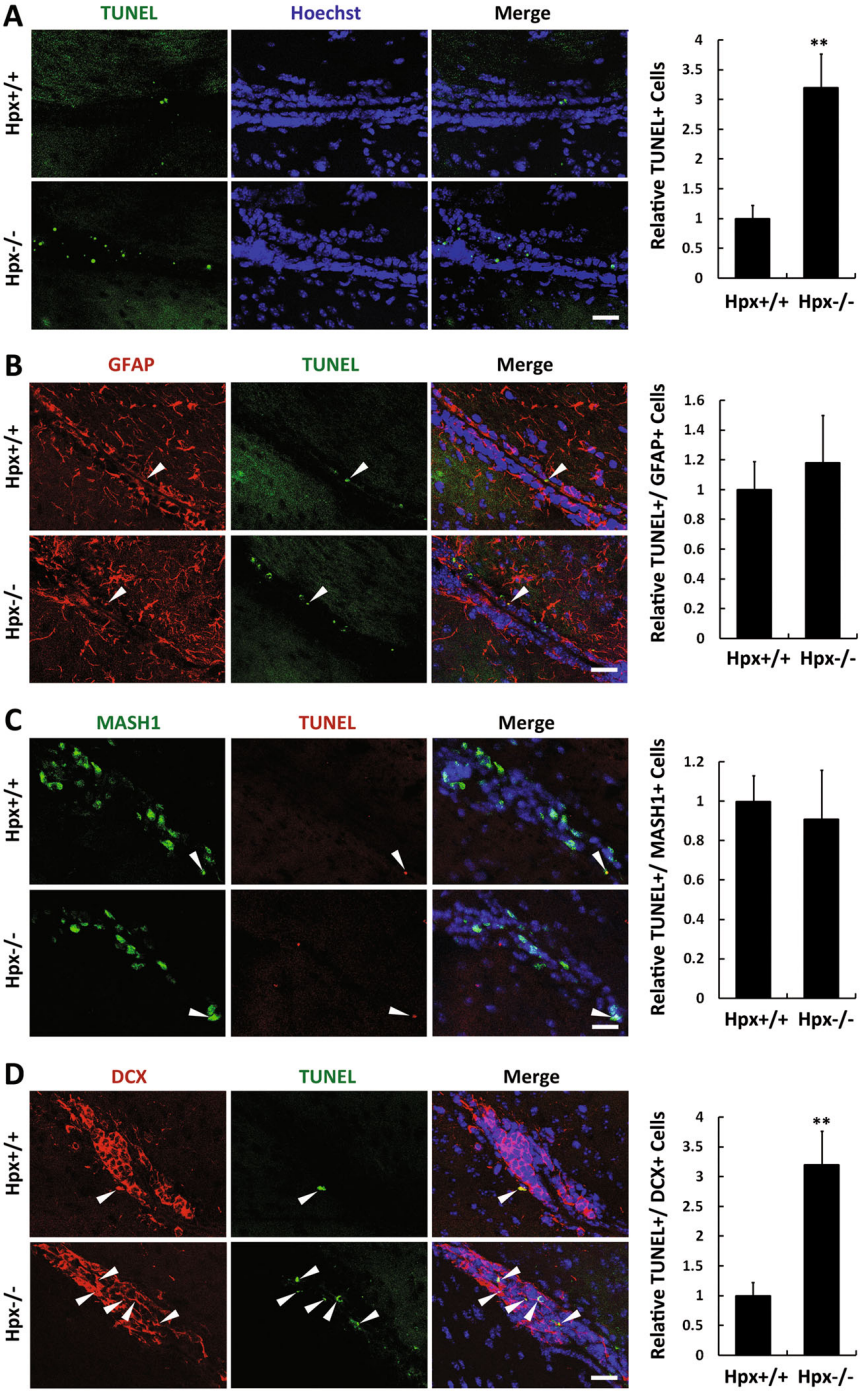
Increased apoptosis in Dcx+ cells but not in GFAP+ or MASH1+ cells in the SVZa of Hpx-null mice. **a** Every fifth coronal section from each brain was subjected to TUNEL (green) assay. Quantification of the total number of TUNEL-labeled cells showed a twofold increase in apoptosis in the SVZa of Hpx^−/−^ mice. **b**–**d** Anti-GFAP (**b**, red), anti-MASH1 (**c**, red), or anti-Dcx (**d**, red) was used for co-labeling (arrowhead) with TUNEL to detect apoptosis in each type of neural stem cells/progenitors. More apoptosis was observed in Dcx+ cells but not in GFAP+ or MASH1+ cells. *n* = 5. **p < 0.01. Scale bar, 20 μm

We next performed TUNEL assays to examine apoptosis in NSCs/progenitors. Quantification of the total number of TUNEL-labeled cells (Fig. 3a) showed a twofold increase in apoptosis in the SVZa of Hpx^−/−^ mice compared to wild-type mice. Because NSCs/progenitors in the SVZ are classified as three major types, different antibodies (anti-GFAP, anti-MASH1, and anti-Dcx) were used as co-labels with TUNEL to detect the types of NSCs/progenitors in which apoptosis increased (Fig. 3b–d). Interestingly, more apoptosis was observed in Dcx+ cells, but not in GFAP+ or MASH1+ cells.

### Hemopexin deletion impairs the migration of neuroblasts to the OB

**Fig. 4:**
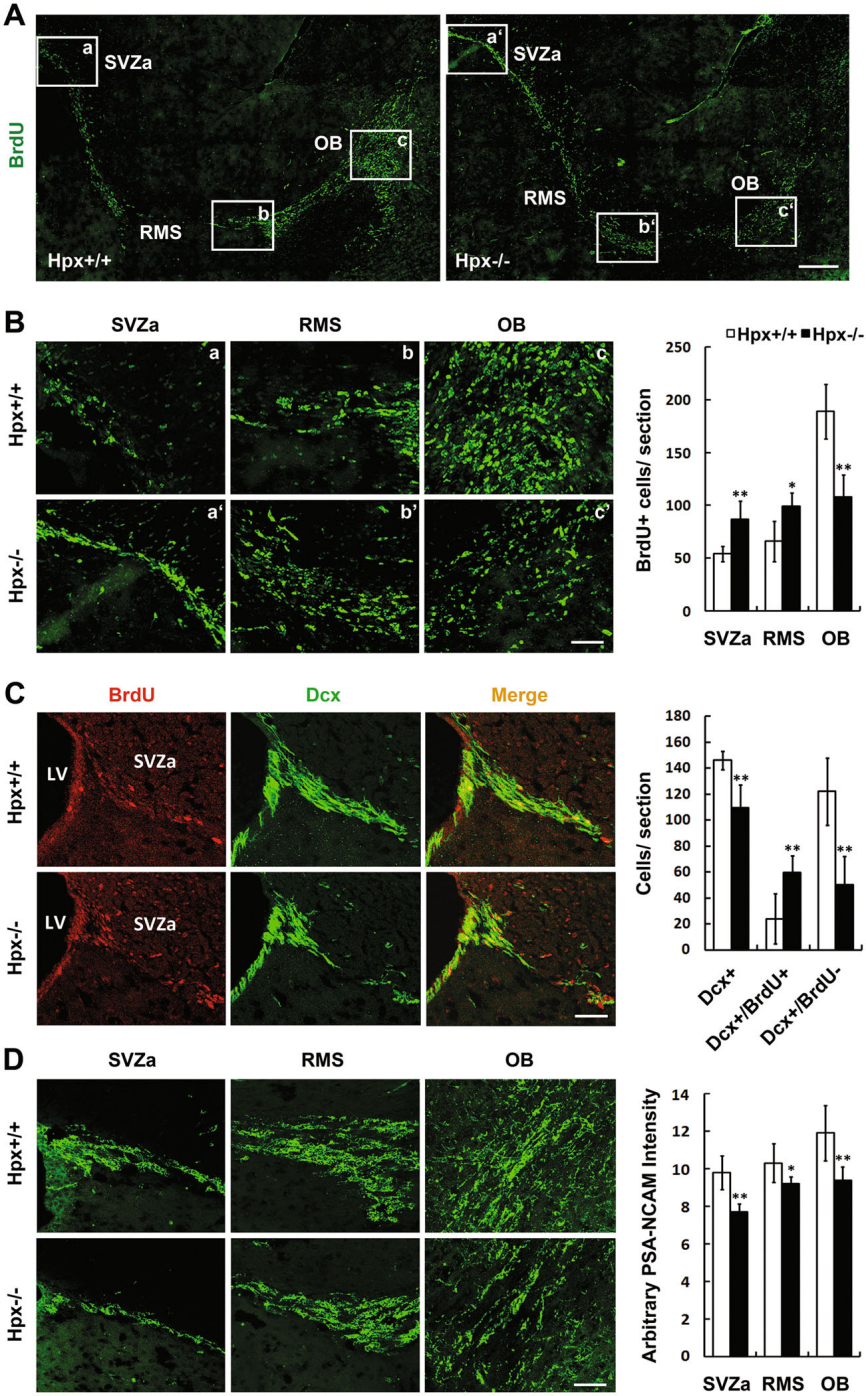
Hemopexin deletion impairs migration of neuroblasts in the RMS. **a** Mice were injected with BrdU for 3 consecutive days and sacrificed 12 days later. Parasagittal sections from each brain were labeled with anti-BrdU (green). **b** High-magnification images showing the BrdU-positive cells in the SVZa, the middle of the RMS, and the core of OB. BrdU+ cells were counted at the three locations in both Hpx^+/+^ and Hpx^−/−^ mice. *n* = 3. **c** Dcx was co-labeled with BrdU staining in the SVZa in parasagittal sections. Dcx+/BrdU+ migrating cells bunched up in the SVZa. *n* = 3. **d** PSA-NCAM staining at three locations in a parasagittal section. Quantification of anti-PSA-NCAM immunostaining intensity showed reduced staining intensity along the RMS in Hpx^−/−^ mice. *n* = 4. *p < 0.05, **p < 0.01. Scale bar, 200 μm in **a**; 50 μm in **b**–**d**. LV lateral ventricle

In the post-natal forebrain, the neuroblasts in the SVZa migrate tangentially along the RMS to the core of the OB, where they migrate to the granule cell layer (GCL) and glomerular layer (GL) and differentiate into mature interneurons. To determine whether the migration of neuroblasts in the RMS in Hpx-deficient mice was perturbed, we examined the distribution of BrdU-labeled cells 12 days after intraperitoneal BrdU injections at three locations along the entire migration pathway: the SVZa, the middle of the RMS, and the core of OB. As shown in Fig. 4a, b, a higher proportion of BrdU-labeled cells stayed in the SVZa and RMS, while a lower proportion reached the OB in the Hpx^−/−^ mice. As reported before, if migration decreases in RMS, then more Dcx+ neuroblasts should have been found bunched up in the SVZa^29,30^. However, as shown in Fig. 1, Dcx+ neuroblasts decreased in SVZa in the Hpx^−/−^ mice. To clarify this problem, we performed the in vivo migration assay as above and co-labeled Dcx+ neuroblasts with BrdU in the sagittal slice of SVZa. As shown in Fig. 4c, although the total Dcx+ cells decreased, Dcx+/BrdU+ cells (which are the labeled migrating cells) bunched up in the SVZa in the Hpx^−/−^ mice, while Dcx+/BrdU− cells decreased in the SVZa.

We further examined immunoreactivity for PSA-NCAM, a marker for migrating neuroblasts, at the three locations along the migration pathway. The pattern of PSA-NCAM immunostaining and the relative orientation of PSA-NCAM+ cells were very similar between WT and Hpx^−/−^ mice (Fig. 4d). However, the PSA-NCAM staining intensity was reduced at the three locations in Hpx^−/−^ mice compared with wild-type mice, suggesting that fewer neuroblasts were migrating in the RMS.

These data indicate that the migration of neuroblasts was impaired in Hpx-deficient mice.

### Hemopexin influence the apoptosis but not proliferation of cultured SVZa stem cells/progenitors

**Fig. 5:**
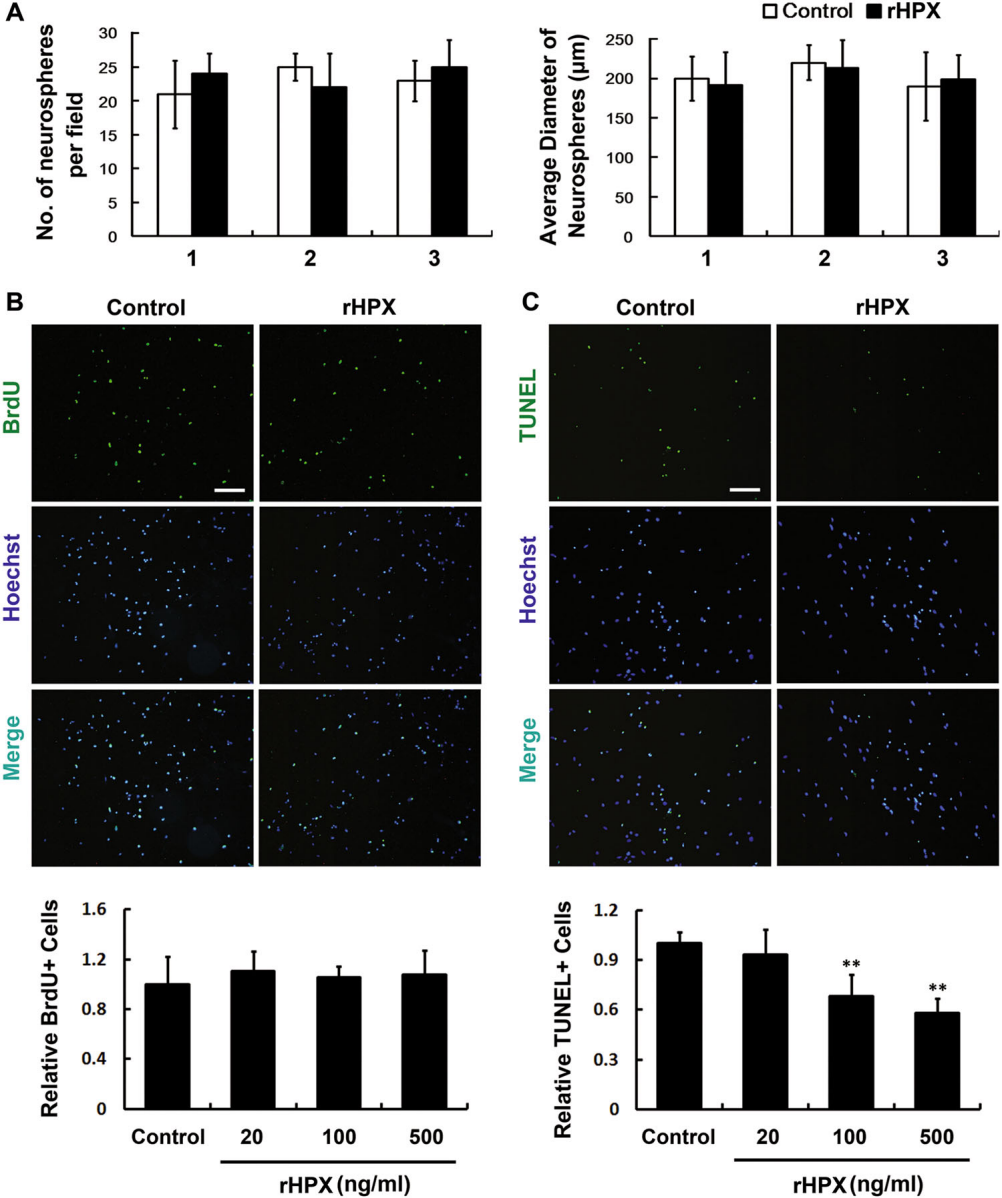
Hemopexin inhibits apoptosis but does not influence proliferation in SVZa stem cells/progenitors in vitro. **a** SVZa neurospheres were dissociated, and single cells were cultured in DM or with recombinant hemopexin (100 ng/ml, rHPX) for 6 days in vitro to enable sphere formation. This course was performed for three passages (1, 2, and 3). Newly formed spheres were examined in each passage, and the average number and diameter of the neurospheres were quantified. **b**, **c** SVZa stem cells/progenitors were cultured in DM or with rHPX for 36 h. BrdU labeling (green) was used to detect proliferation **b**, and TUNEL staining (green) was used to detect apoptosis **c**. The percentage of BrdU+ or TUNEL+ cell numbers was calculated in each group. *N* = 3. **p < 0.01 versus control. Scale bar: 100 µm

To further clarify the role of Hpx, we studied proliferation and apoptosis in cultured SVZa stem cells/progenitors, using the recombinant Hpx protein (rHPX, R&D). In the neurosphere formation assay, no significant change was observed in either the number or the diameter of newly formed neurospheres in all three consecutive passages (Fig. 5a). And no difference was detected in the number of BrdU+ cells following exposure to any concentration of rHPX in the BrdU assay (Fig. 5b). Correspondingly, decreasing in the numbers of TUNEL-labeled cells was induced by increasing concentrations of rHPX (Fig. 5c). Since the recombinant forms of Hpx remain essentially uncharacterized, it was queried if these rHPX are properly folded, or if it can bind heme because its conformation may be changed. So, we also tried the native Hpx purified from human plasma (nHPX, Athens Research & Technology), which was proved to bind heme correctly. As shown in Supplemental Fig. S1, the purified nHPX could also inhibit apoptosis of cultured SVZa stem cells/progenitors with stronger effect. These data suggesting that Hpx inhibits apoptosis but does not influence the proliferation of in SVZa stem cells/progenitors in vitro.

### Hemopexin promotes the migration of cultured SVZa stem cells/progenitors

**Fig. 6:**
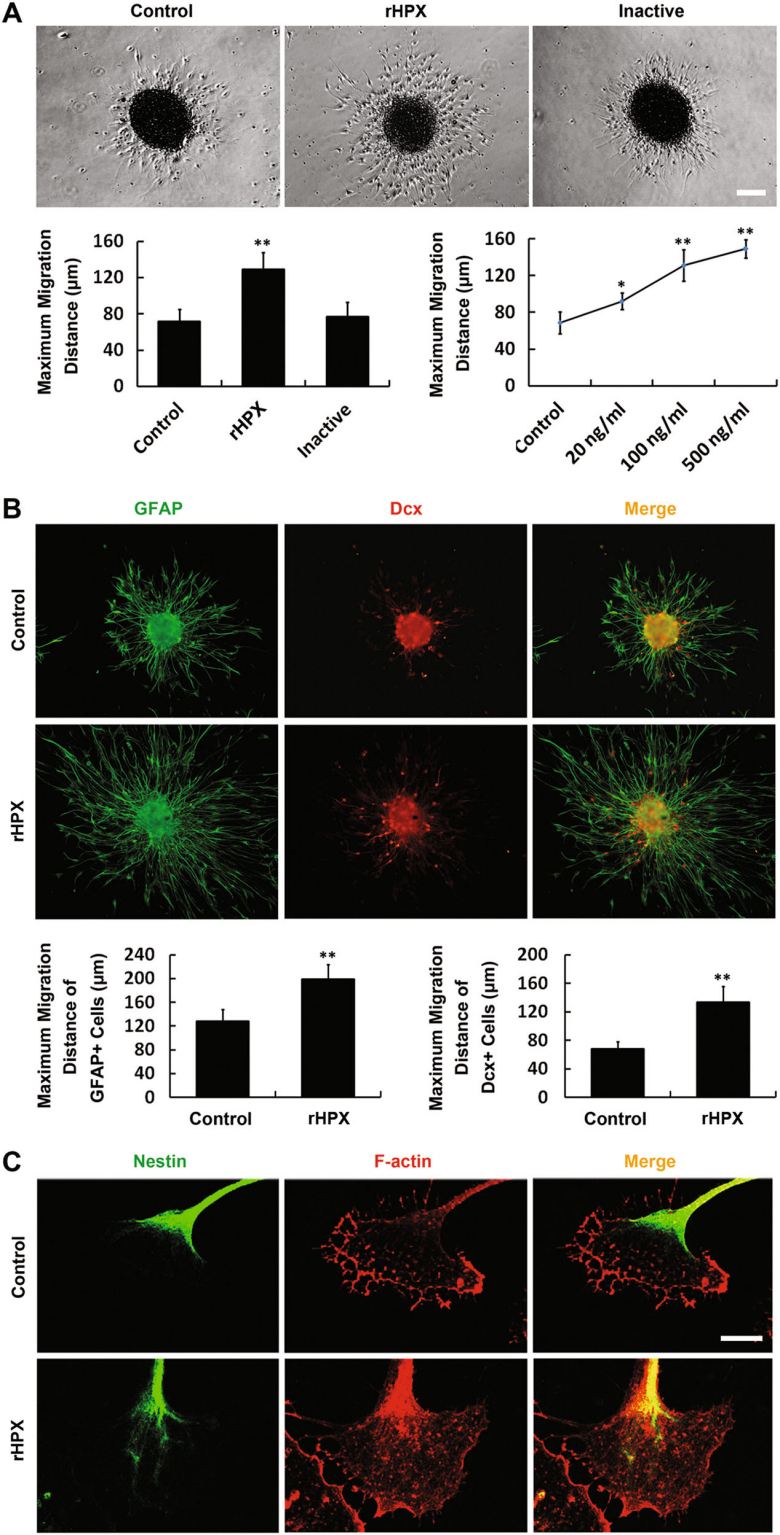
Hemopexin promotes cultured SVZa stem cells/progenitors migration and induces cytoskeletal reorganization in vitro. **a** SVZa neurospheres were cultured with or without rHPX for 36 h. Inactive hemopexin was used as a negative control. The mean maximum migration distance in each group was calculated. **b** SVZa neurospheres were cultured with or without rHPX (100 ng/ml) for 36 h. Cells were labeled with anti-GFAP (green) and anti-Dcx (red). The mean maximum migration distance of GFAP+ and Dcx+ cells was calculated, respectively. **c** SVZa stem cells/progenitors were cultured in DM or with rHPX for 36 h. Cells were labeled with Nestin (green) and F-actin (red). *N* = 3. *p < 0.05, **p < 0.01 versus control. Scale bar: 50 µm in **a** and **b**; 5 µm in **c**

To further investigate the role of Hpx in the motility of NSCs/progenitors, we performed in vitro migration assays. As shown in Fig. 6a, NSCs/progenitors migrated further in the rHPX group than in the control or the inactive Hpx group. When neurospheres were cultured with increasing amounts of rHPX, the mean maximum migration distance increased according the increased concentration of rHPX. To clarify which kinds of cells were affected in the in vitro migration assay, anti-GFAP (green) and anti-Dcx (red) were used to label stem cells and neuroblasts, respectively. The mean maximum migration distance of each cell type was calculated. As shown in Fig. 6b, although most cells migrated out of the neurospheres were GFAP+ cells, both GFAP+ and Dcx+ cells migrated further after adding the rHPX. The purified nHPX was also proved to promote the migration of cultured SVZa stem cells/progenitors (Supplemental Fig. S2).

Because the reorganization of the cytoskeleton is essential for cell motility^31^, we next examined whether this improved migration could be attributed to changes in the distribution of actin filaments. Cultured NSCs/progenitors were co-labeled with anti-Nestin and F-actin. Increased F-actin was observed in the peripheral domains of lamellipodial cytoskeletons in the Nestin+ cells after Hpx stimulation (Fig. 6c).

These combined results demonstrate that Hpx promotes cultured SVZa stem cell/progenitors migration and induces cytoskeletal reorganization in vitro.

### Hemopexin influences the differentiation of cultured SVZa stem cells/progenitors

**Fig. 7:**
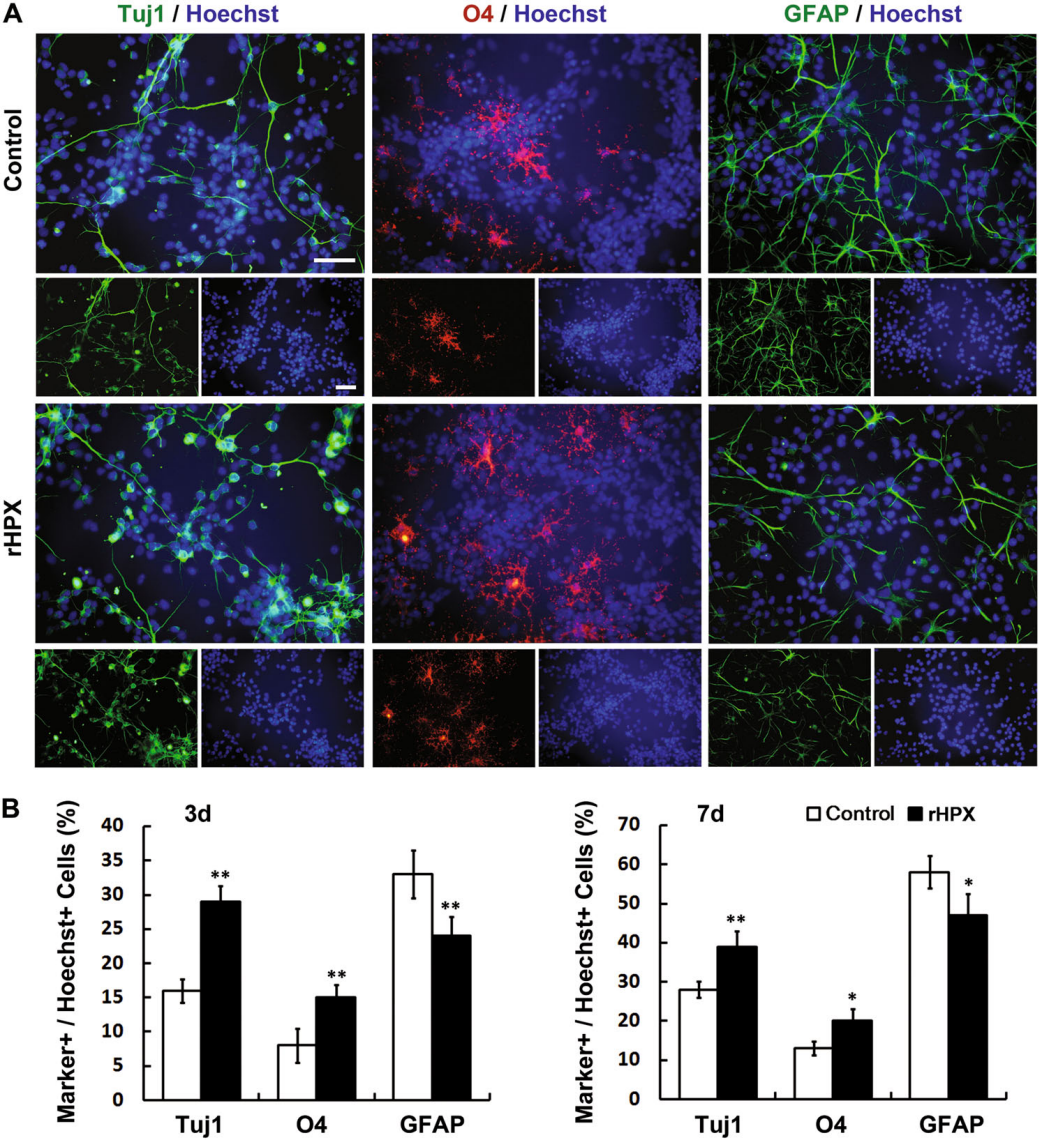
Hemopexin promotes cultured SVZa stem cells/progenitors to differentiate into neurons and oligodendrocytes in vitro. **a** SVZa stem cells/progenitors were cultured in DM or with rHPX (100 ng/ml) for 3 days. Cells were labeled with Tuj1 (green), O4 (green), or GFAP (red). Hoechst (blue) was used to label nuclei. **b** The percentage of Tuj1+, O4+, and GFAP+ cells was calculated in each group after cells were cultured with rHPX for 3 days or 7 days, respectively. More Tuj1+ and O4+ cells but fewer GFAP+ cells were observed in the rHPX group. *N* = 3. *p < 0.05, **p < 0.01. Scale bar: 50 µm

In the RMS, neuroblasts keep differentiating while migrating toward the OB, and they finally differentiate into different subtypes of interneurons in the OB^6,32^. To further explore the effect of Hpx on the differentiation of NSCs, we examined the expression of Tuj1 (neuron marker), O4 (oligodendrocyte marker), and GFAP (astrocyte marker) in cultured SVZa stem cells by immunostaining. Compared with the control group, the percentage of Tuj1+ and O4+ cells increased, while the percentage of GFAP+ cells decreased in the rHPX group (Fig. 7). The purified nHPX was proved to have the same effect as the rHPX did (Supplemental Fig. S3). These results indicate that Hpx promotes cultured SVZa stem cells to differentiate into neurons and oligodendrocytes in vitro.

### Hemopexin deletion impairs the genesis of interneurons in the OB

The final fate of SVZa NSCs is to reach the OB and differentiate into interneurons^6^. To explore the morphologic change of the OB in Hpx^−/−^ mice, we examined the shape and measured the size of the brain and the OB (Supplemental Fig. S4). No significant difference was found in the shape of brain, telencephalon, or OB between wild-type and Hpx-deficient mice. In addition, no significant difference was observed in the volume of the OB.

**Fig. 8:**
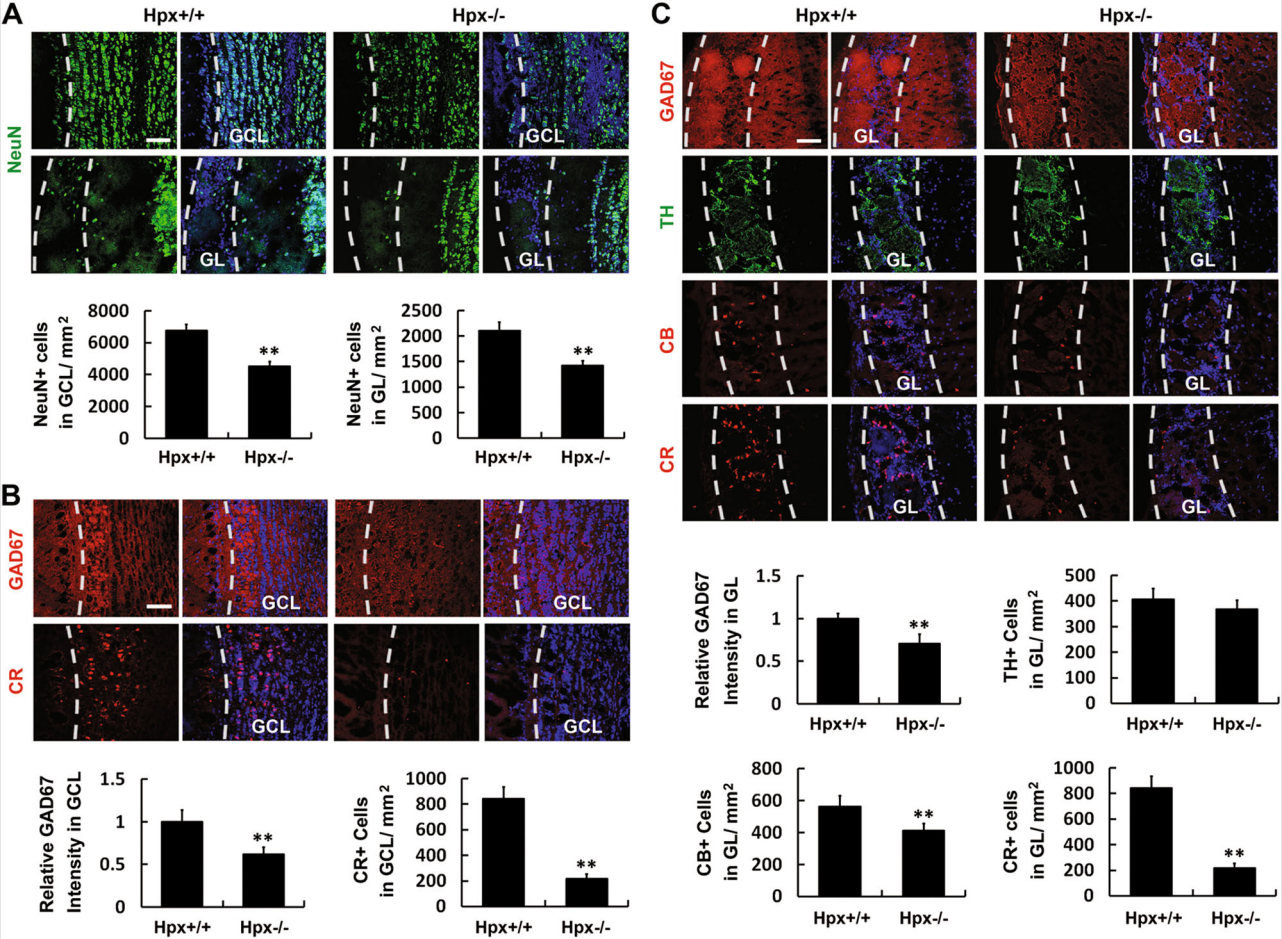
Hemopexin deletion impairs the genesis of interneurons in the OB. Coronal sections of the OB of Hpx^+/+^ and Hpx^−/−^ mice were analyzed. **a** Anti-NeuN immunostaining (green) was performed to label the neurons. Hoechst staining (blue) was used to identify all nuclei and overall structure. Hemopexin deletion reduced the density of NeuN+ neurons in both the GCL and the GL of the OB. **b** Anti-GAD67 (red) and anti-CR (red) were used to label GABAergic neurons and CR+ interneurons, respectively, in the GCL of the OB. Immunoreactivity for GAD67 and the cell density of CR+ cells in the GCL were significantly reduced in Hpx^−/−^ mice. **c** Anti-GAD67 (red), anti-TH (green), anti-CB (red), and anti-CR (red) were used to label GABAergic neurons, dopaminergic neurons, CB+ interneurons and CR+ interneurons, respectively, in the GL of the OB. Immunoreactivity for GAD67 and the cell density of calbindin+ (CB+) and calretinin (CR+) interneurons were decreased in the GL in Hpx^−/−^ mice, while the cell density of TH+ dopaminergic neurons in the GL was not affected. *n* = 5. **p < 0.01. Scale bar: 100 μm. GCL granule cell layer, GL glomerular layer

To clarify the effect of Hpx on the final fate of SVZa stem cells/progenitors, coronal sections of OB from Hpx^+/+^ and Hpx^−/−^ mice were examined by immunostaining with an antibody against NeuN, a neuron marker, in the GCL and GL. As shown in Fig. 8a, the density of NeuN+ neurons was significantly reduced in both the GCL and GL of the OB in Hpx^−/−^ mice compared to wild-type mice, suggesting that Hpx deletion reduces the number of interneurons in the OB.

The inhibitory interneurons in the GCL of the OB are all GABAergic, while the periglomerular neurons (a population of interneurons surrounding the glomeruli) are heterogeneous^33,34^. To examine changes in each subtype of interneurons in the OB, anti-GAD67 and anti-CR were used to label GABAergic neurons and calretinin+ (CR+) interneurons in the GCL; while anti-GAD67, anti-TH, anti-CB, and anti-CR were used to label GABAergic neurons, dopaminergic neurons, CB+ interneurons, and CR+ interneurons in the GL, respectively (Fig. 8b, c). Hemopexin deletion significantly reduced the immunoreactivity for GAD67 in both the GCL and the GL of the OB, as well as the cell density of CR+ cells in the GCL and the cell density of CB+ and CR+ interneurons in the GL. However, the cell density of TH+ dopaminergic neurons in the GL was not changed. Taken together, these data indicate that the genesis of OB interneurons is impaired in Hpx-deficient mice.

## Discussion

The NSCs/progenitors in the SVZ lie within a special niche, and factors in this niche are critical for neurogenesis^5^. In this study, we show that Hpx, which is widely known to be a heme scavenger at the systemic level^22^, is an important factor involved in neurogenesis in the SVZ/OB pathway (Supplemental Fig. S6). Our results not only reveal a previously unknown function for the protein Hpx in the CNS, but also suggest Hpx as a new regulatory factor during adult neurogenesis.

Hemopexin is a plasma glycoprotein produced mainly by the liver^35^. It has also been described in the ependymal cells that line the ventricular system^23^ and in CSF^24^. Thus, ependymal cells, plasma, and CSF may be sources of the Hpx that contributes to SVZ neurogenesis. In this study, we verified that ependymal cells express Hpx in wild-type mice (Fig. S5A, B). After knockout of Hpx, no change was found in the number of ependymal cells compared to Hpx+/+ mice, nor the alinement of ependymal cells. These data suggest that knockout of Hpx did not cause any apparent ependymal phenotype. However, further researches were needed to clarify whether the phenotype of neurogenesis in the SVZ was direct or indirect after Hpx knockout.

The SVZ is one of the most important regions for neurogenesis in the adult mammalian brain^1,2^. Abnormal changes in cell proliferation or apoptosis in this stem cell pool can influence the size of the lateral ventricles^27,28^. In the present study, enlargement of the lateral ventricles was observed in Hpx-deficient mice. In the BrdU assay, we did not detect any difference on the proliferation of SVZ neural precursors between Hpx-deficient and wild-type mice. However, TUNEL assays revealed that more apoptosis occurred in Dcx+ cells in the SVZa in Hpx-null mice, and in vitro assays showed that exogenous Hpx inhibited apoptosis but did not influence the proliferation of cultured SVZa stem cells/progenitors. Increased apoptosis may result in a reduction in the total number of cells in the SVZa. This may be one of the reasons for the enlargement of the lateral ventricles observed in Hpx-deleted mice. Moreover, it is widely accepted that neuroblasts generated in the SVZ not only migrate to the OB, but also migrate into areas that are not normally neurogenic, for example, the striatum and cerebral cortex^36–38^. Increased apoptosis of SVZ stem cells may also lead to a reduction in the total number of cells in these areas, which then result in a thinner cerebral cortex and the enlargement of the lateral ventricles. To clarify this, further researches were needed.

The neuroblasts in the SVZ migrate anteriorly in chains of cells along the RMS to the OB^3,39^. In the present study, we showed that the PSA-NCAM staining intensity was reduced along the RMS in Hpx^−/−^ mice compared to wild-type mice. This may be due to decreased cell numbers in the RMS because inhibited migration and increased apoptosis of Dcx+ neuroblasts was detected. However, the migration assays combined with BrdU labeling showed that a higher proportion of BrdU-labeled cells stayed in the SVZa and RMS, while fewer cells reached the OB in Hpx-null mice. One may ask that if migration decreases in vivo in the Hpx^−/−^ mice, then more Dcx+ neuroblasts should have been found bunched up in the SVZa. However, as shown in Fig. 1, Dcx+ neuroblasts decreased in SVZa in the Hpx^−/−^ mice, so did the data in Fig. 4c. To clarify this problem, the composition of Dcx+ cells in the SVZa should be analyzed. In the mice used for the in vivo migration assay who had intraperitoneal BrdU injections 12 days ago, the total Dcx+ cells in the SVZa were composed of two kind of cells: Dcx+/BrdU+ cells (which are the BrdU-labeled migrating neuroblasts 12 days ago) and Dcx+/BrdU− cells (which may come from GFAP+ cells between the 12 days). As shown in Fig. 4c, Dcx+/BrdU+ cells bunched up in the SVZa in the Hpx^−/−^ mice, indicating that the migration of neuroblasts was inhibited. While Dcx+/BrdU− cells decreased in the SVZa, this may be caused by inhibited lineage differentiation from GFAP+ cells toward Dcx+ cells and increased apopsis of Dcx+ cells. Moreover, in vitro migration assays demonstrated that Hpx enhanced the migration of stem cells/progenitors from the neurospheres. We also evaluated ectopic migration of Dcx+ neuroblasts in the RMS, but no statistical difference was observed. Taken together, these results support the notion that the migration of neuroblasts in the RMS was impaired after Hpx deletion.

Until recently, the only known receptor for Hpx is LRP1^40^. Previous studies demonstrated that LRP1 is expressed in neurons, perivascular astrocytes, microglial cells, epithelial cells of choroid plexi, and endothelial cells of microvessels in CNS^41^. We performed an immunocytochemistry assay and found that it is also expressed in the cultured SVZ stem cells (Supplemental Fig. S5C), supporting the possibility that Hpx may influence the neurogenesis in the SVZ by interacting with LRP1. LRP1 is a scavenger receptor for an array of ligands, whose function is thought to endocytose the ligands from the membrane and deliver them to lysosomes for degradation^42^. As the receptor for Hpx, LRP1 was reported to mediate the heme-Hpx internalization, resulting in cellular heme uptake^40^. However, in our in vitro studies (Figs. 5–7), the exogenous Hpx protein inhibited apoptosis but promoted migration and differentiation of cultured NSCs in an environment free of heme. This suggested that these effects of Hpx may be due to the apo-protein but not the heme-Hpx complex. As reported, apo-Hpx can also bind LRP1, although not so strong as heme-Hpx^40^. And it is noteworthy that LRP1 can interact with scaffolding and signaling proteins via its intracellular domain in a phosphorylation-dependent manner, which may trigger receptor-activation and -signaling pathways^42^, and thus promote cell migration^43,44^, inhibit apoptosis^45^, and influence differentiation^46^. So, it is possible to speculate that Hpx acts in a similar manner by activating LRP1 in the NSCs and recruiting different adapter molecules, thereby triggering appropriate intracellular signals and controlling cell survival, migration, and lineage differentiation.

Neurogenesis of SVZ stem cells is important for the development and functional maintenance of the OB^47^. There is evidence suggesting that ongoing neurogenesis could provide for the replacement of interneurons that are lost through programmed cell death in the OB^48^. A continuous supply of new neurons may allow for the maximization of odor discrimination, adaptation to changing environmental conditions, and the renewing of memories^49^. Olfactory dysfunction is involved in various neurologic and neurodegenerative diseases^50–52^. Endogenous neurogenesis has been found to be altered in the SVZ of patients with AD, who show a significant reduction in undifferentiated neural progenitors but an increase in GFAP-negative astrocyte-like cells with progenitor characteristics^53,54^. Abnormalities in adult neurogenesis in PD patients have also been described^55,56^, and the decrease in the number of EGF receptor-positive cells in the adult SVZ in human PD patients has been shown to be an indirect sign of reduced OB neurogenesis^57^. Our finding that Hpx, as an extrinsic factor, can influence adult neurogenesis by regulating the apoptosis, migration, and differentiation of NSCs/progenitors in the SVZ not only suggests a new molecular mechanism for the adult stem cell niche that regulates neurogenesis, but also may benefit the understanding for olfactory system development and related neurodegenerative diseases.

## Materials and methods

### Animals

All animal experiments were performed in accordance with the National Institutes of Health Guidelines on the Use of Laboratory Animals and approved by the Second Military Medical University Committee on Animal Care.

The Hpx knockout mice (Hpx^−/−^) used in this study were developed at the South Carolina Research Foundation, USA, and transferred by Dr. Raymond F. Regan at Thomas Jefferson University, USA. Mice were bred with wild-type B6; 129 mice that were maintained in our colony. To minimize genetic differences between the wild-type and knockout groups, heterozygotes were used for breeding. Genotype was determined by PCR of genomic DNA that was extracted from proteinase K-treated tail clippings. The following three primers were used:

Hpx wild-type and knockout, forward: 5′-TCCTGTGTGGCCTTTGCAGC-3′.

Hpx wild-type, reverse: 5′-CAACTTCGGCAACTCTCCCG-3′.

Hpx knockout, reverse: 5′-GATGCGGTGGGCTCTATGGC-3′.

### Tissue processing and immunohistochemistry

Ten-week-old adult Hpx +/+ and Hpx^−/−^ littermates were used for the in vivo assay. Mice were anesthetized with 100 mg/kg sodium pentobarbital and then perfused transcardially with PBS followed by 4% PFA/PBS. Brains with OB were removed and post-fixed in 4% PFA/PBS overnight at 4 °C and then cryo-protected in a gradient of sucrose to 30% (w/v). Brains were then embedded in Tissue-Tek OCT compound (Sakura). The brains were stored at −80 °C until sectioned. Sections of 14 μm thickness were obtained in the coronal and sagittal planes using a Leica cryostat and collected onto Superfrost Plus slides (Fisher Scientific). Slides were then air-dried for 1 h before being stored at −80 °C until staining.

Frozen sections were rinsed with PBS for 10 min and then permeabilized with 0.4% Triton X-100 in 0.1 M PBS for 60 min. The sections were then blocked with PBS containing 0.4% Triton X-100 and 10% normal donkey serum for 1.5 h at room temperature and then incubated overnight at 4 ℃ with antibodies to GFAP (1:200, Sigma), MASH-1 (1:100, BD Biosciences), doublecortin (1:1000, Abcam), PSA-NCAM (1:200, Chemicon), NeuN (1:100, Chemicon), TH (1:2000, Sigma), GAD67 (1:200, Chemicon), calretinin (CR, 1:500, Chemicon), or calbindin (CB, 1:500, Chemicon) diluted in PBS containing 10% normal goat serum. After washing three times with 0.1 M PBS (pH 7.4), cells were incubated with a FITC- or TRITC-conjugated secondary antibody (Jackson Immuno Research Laboratories, Inc., West Grove, PA) for 2 h at room temperature. Cell nuclei were labeled with 1 μg/ml Hoechst 33342 (Sigma). Finally, brain sections were mounted with anti-fade Aqua Poly/Mount (Polysciences).

### Neurosphere culture and formation assay

Neurospheres were prepared from SVZa explants dissected from neonatal Sprague-Dawley rats^58^. In brief, brains were dissected and embedded in 4% low melting point agarose prepared in PBS. Sagittal sections of 250 μm were cut on a vibratome (Vibratome 3000). Tissues from the SVZa were isolated, chopped, and digested with 0.125% trypsin at 37 °C for 20 min and then washed with FBS-containing medium, triturated gently, and centrifuged for 5 min at 1000 r.p.m. The collected cells were re-suspended at a density of 3 × 10^4^ cells per ml and cultured in defined medium (DM), that is serum-free DMEM/F12 medium supplemented with B-27 (1:50), bFGF (10 ng/ml, Sigma), and EGF (10 ng/ml, Sigma). Neurospheres were passaged three times to remove any contaminating neurons and other cells.

Neurospheres were dissociated and single cells were cultured in suspension in DM or with Hpx protein (100 ng/ml, recombinant human hemopexin protein, R&D, 4490-HP; purified hemopexin from human plasma, Athens Research & Technology, 16-16-080513) for 6 days in a culture flask (25 cm^2^) to enable sphere formation. This protocol was performed for three passages. Newly formed spheres were examined for each passage, and the average number and diameter of neurospheres were quantified as described previously^59,60^.

### Immunocytochemistry

Neurospheres were dissociated as indicated above, and the collected cells were re-suspended in DMEM/F12 supplemented with B-27, bFGF, and EGF at a density of 3 × 10^4^ cells per ml. Cells were then plated onto PLL-coated 24-well culture dishes (Costar) at 1 ml per well. Hemopexin protein was added after adherence for 24 h in BrdU/TUNEL assays and for 3 and 7 days in differentiation assays.

Immunocytochemistry was performed as described previously^61^. The cell preparations were fixed in 4% paraformaldehyde at 4 °C for 20 min and then washed in PBS three times. The preparations were then treated with 2 M HCl for 15 min, 0.1 M sodium borate for 15 min, and 0.4% Triton X-100 for 10 min at room temperature. Next, nonspecific binding sites were blocked with 10% normal donkey serum in PBS. The preparations were then successively incubated in primary antibodies overnight at 4 °C, and then with a FITC- or TRITC-conjugated secondary antibody for 1 h. The primary antibodies used were as follows: Tuj1 (1:1000, Promega), O4 (1:100, Chemicon), and GFAP (1:200, Sigma). Cell nuclei were labeled with Hoechst 33342.

### BrdU assay

Bromodeoxyuridine (5′-bromo-2′-deoxyuridine; BrdU) (Sigma) was dissolved in 0.9% sodium chloride/0.007% NaOH. To examine proliferation, the mice received one intraperitoneal injection of BrdU (100 mg/kg) 2 h before perfusion and killing^62,63^. To examine migration, another cohort received three intraperitoneal BrdU injections per day for 3 consecutive days, and these mice were then killed 12 days after the last injection^63^. Immunohistochemical detection of BrdU was performed as previously described with slight modification^62^. Briefly, brain sections were first subjected to an HCl antigen retrieval method by incubation in 2 N HCl for 1 h at 37 °C and then neutralization by rinsing three times for 5 min each in 0.1 M pH 8.5 borate buffer. Sections were then subject to immunohistochemical staining for BrdU. For cell assays in vitro, neurospheres were dissociated and single cells were seeded and cultured with increasing amounts of Hpx for 36 h. BrdU (10 μM) was added for 6 h before sections were fixed in 4% paraformaldehyde, and an anti-BrdU (1:500) antibody was used to detect proliferation. The percentage of BrdU+ cells was calculated in each group.

### TUNEL assay

TUNEL assays (Terminal-deoxynucleoitidyl Transferase Mediated Nick End Labeling) were performed according to the manufacturer’s instructions for the In Situ Cell Death Detection Kit, TMR red (Roche). The total number of TUNEL+ cells and the ratio of TUNEL-labeled cells versus GFAP/MASH1/Dcx-labeled cells in the area of the SVZa were calculated. For cell assays in vitro, SVZa stem cells/progenitors were cultured with increasing amounts of Hpx for 36 h. The percentage of TUNEL+ cells was calculated in each group.

### Migration assay in vitro

Migration assays in vitro were performed as described in our previous methods^58^. Briefly, floating neurospheres were transferred onto PLL-coated 24-well culture dishes (Costar) and cultured with or without Hpx for 36 h at 37 °C. Then, neurospheres were fixed with 4% PFA, and cell nuclei were labeled with Hoechst 33342. The maximum distance between the leading cells and the edge of the neurospheres was measured.

### Differentiation assay in vitro

SVZa neurospheres were dissociated, and single cells were seeded and cultured in DM or with Hpx (100 ng/ml). Hemopexin was added every 2 days. After being cultured for 3 days or 7 days, cells were fixed and labeled with anti-Tuj1, anti-O4, or anti-GFAP antibodies. The percentage of Tuj1+, O4+, or GFAP+ cells was calculated in each group.

### Image capturing and quantification

All images were captured with a confocal microscope (Leica SP5). ImageJ software was used to quantify immunostaining intensity. For the in vivo assay, every fifth serial coronal section was used for cell counting, while every third parasagittal section was used for cell counting. For each section, the region of the SVZ, RMS, or OB was captured under the ×40 lens and outlines were determined by Hoechst staining. Cell counting was performed in the SVZa, RMS or in the dorsal, medial, lateral, and ventral OB under the ×40 objective with the counting frame set at 50 × 50 um^2^. At least 1000 cells were sampled for each brain region per animal.

The stereological quantification of the volume of the lateral ventricles was performed as previously reported^62^. Every fifth coronal section (between 0.10 and 1.18 mm from the bregma) that was stained with Hoechst was used for measurements. The volume of the lateral ventricles was approximated by using the equation ∑ (*i* = 0 to *n*)*A*_*i*_ *d*, where *A* equals the area of the *i*^th^ section, *d* is the distance between sections, and *n* is the total number of measured sections.

### Statistical analysis

All data represent the results of at least three independent experiments. Pairwise comparison of the means was analyzed by Student’s *t* test, two-tailed analysis. One-way analysis of variance was used to analyze in vivo data comparing across the Hpx +/+ and Hpx^−/−^ mice. All data are presented as the mean ± SD.

## Electronic supplementary material

Supplemental Information
