# Supplementary material for: Hemopexin is required for adult neurogenesis in the subventricular zone/olfactory bulb pathway
Source: Cell Death Dis. 2018 Feb 15;9(3):268. doi: 10.1038/s41419-018-0328-0 (PMC5833796; doi:10.1038/s41419-018-0328-0)
Supplement: Supplementary file 1 — Supplemental Information [file 41419_2018_328_MOESM1_ESM.doc]

**Supplementary Information**

**Supplementary Figures and Legends**

**
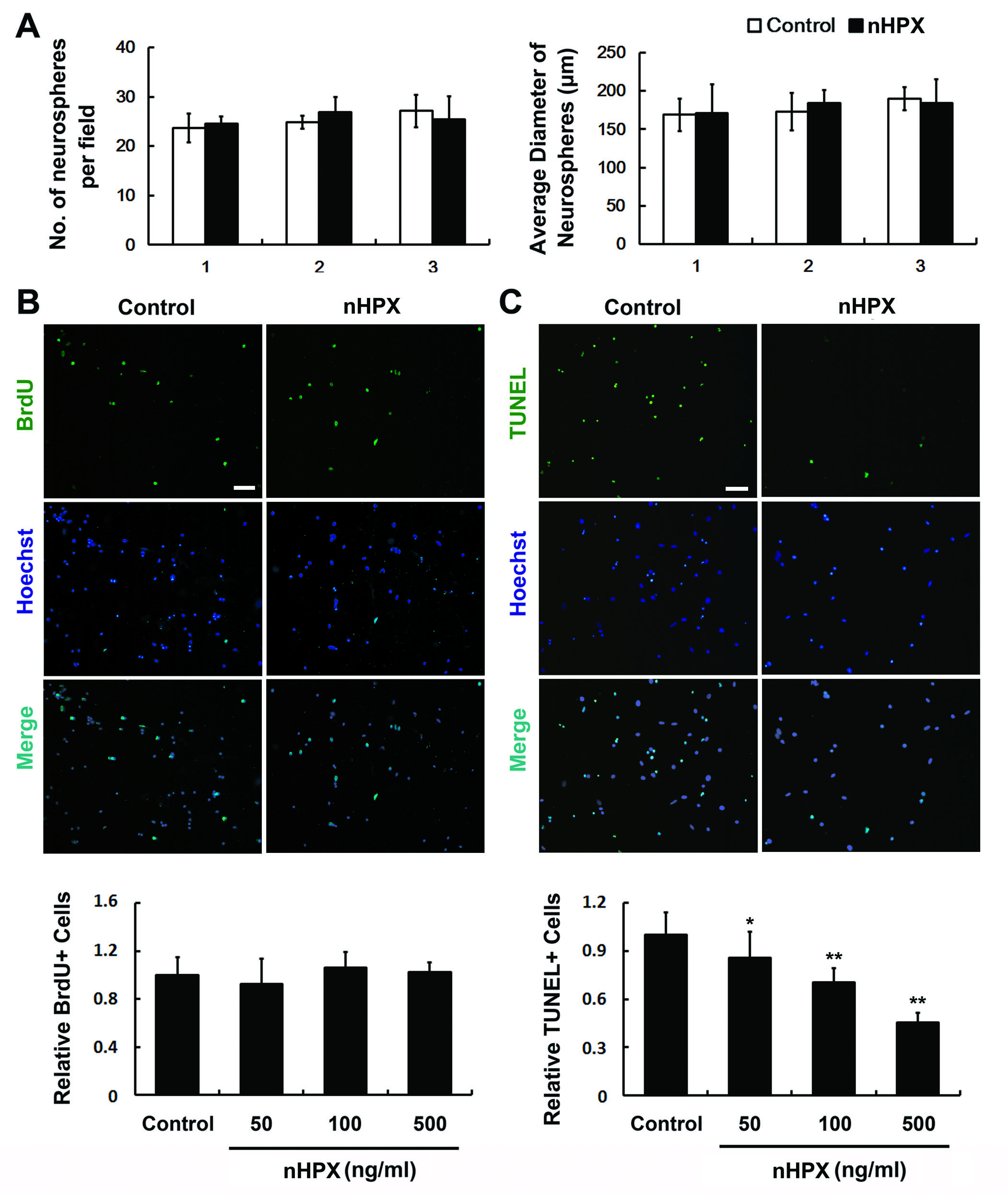
**

**Figure S1.** Hemopexin inhibits apoptosis but does not influence proliferation in SVZa stem cells/ progenitors in vitro. (A) SVZa neurospheres were dissociated, and single cells were cultured in DM or with native hemopexin (nHPX) for 6 days in vitro to enable sphere formation. This course was performed for three passages (1, 2 and 3). Newly formed spheres were examined in each passage, and the average number and diameter of the neurospheres were quantified. (B, C) SVZa stem cells/ progenitors were cultured in DM or with nHPX for 36 h. BrdU labeling (green) was used to detect proliferation (B), and TUNEL staining (green) was used to detect apoptosis (C). The percentage of BrdU+ or TUNEL+ cell numbers was calculated in each group. N=3. *p<0.05, **p<0.01 versus control. Scale bar: 50 µm.

**
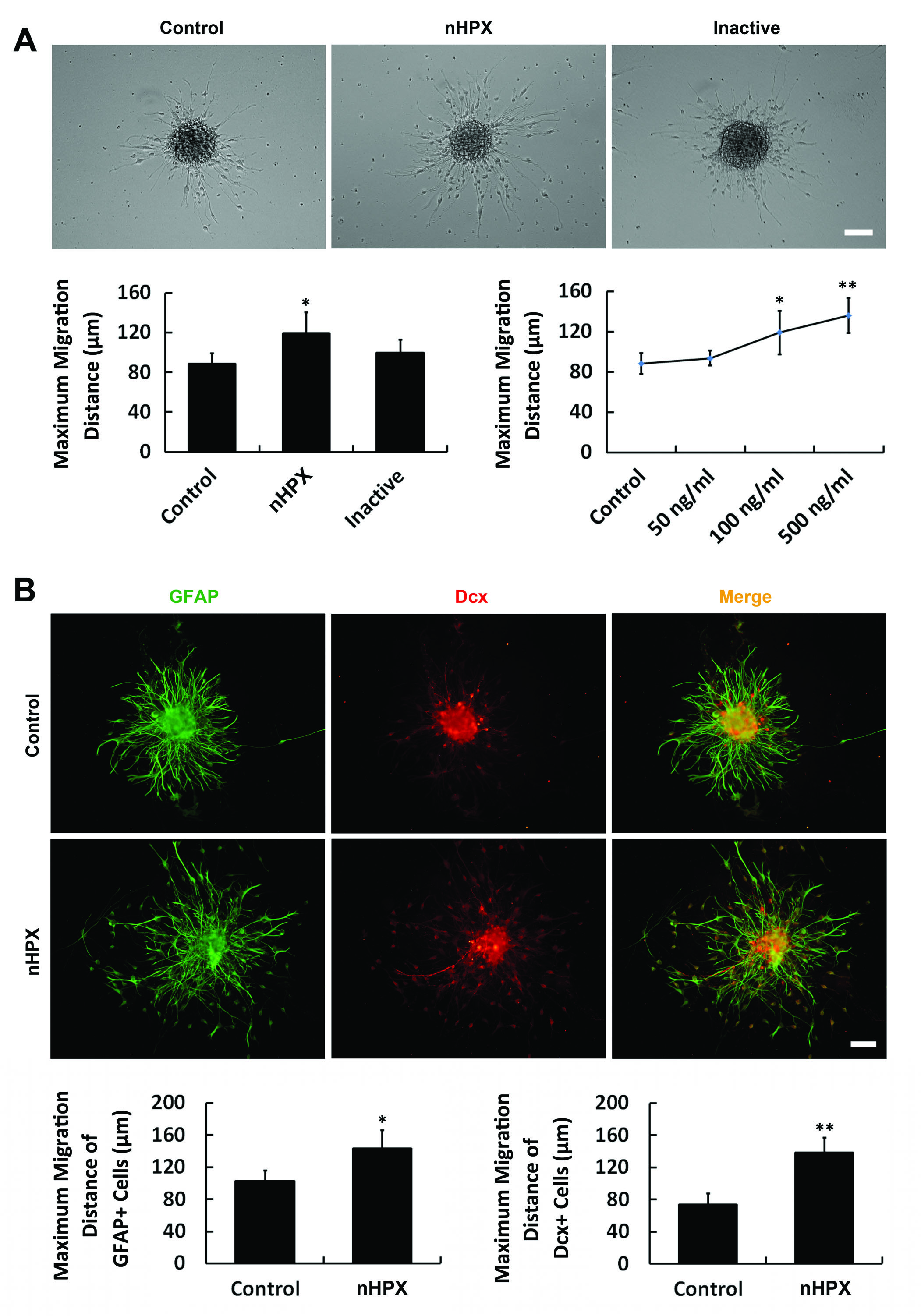
**

**Figure S2.** Hemopexin promotes cultured SVZa stem cells/ progenitors migration and induces cytoskeletal reorganization in vitro. (A) SVZa neurospheres were cultured with or without nHPX for 36 h. Inactive hemopexin was used as a negative control. The mean maximum migration distance in each group was calculated. (B) SVZa neurospheres were cultured with or without nHPX (100ng/ ml) for 36 h. Cells were labeled with anti-GFAP (green) and anti-Dcx (red). The mean maximum migration distance of GFAP+ and Dcx+ cells were calculated, respectively. (C) SVZa stem cells/ progenitors were cultured in DM or with nHPX (100ng/ml) for 36 h. Cells were labeled with Nestin (green) and F-actin (red). N=3. *p<0.05, **p<0.01 versus control. Scale bar: 50 µm in A and B; 5 µm in C.

**
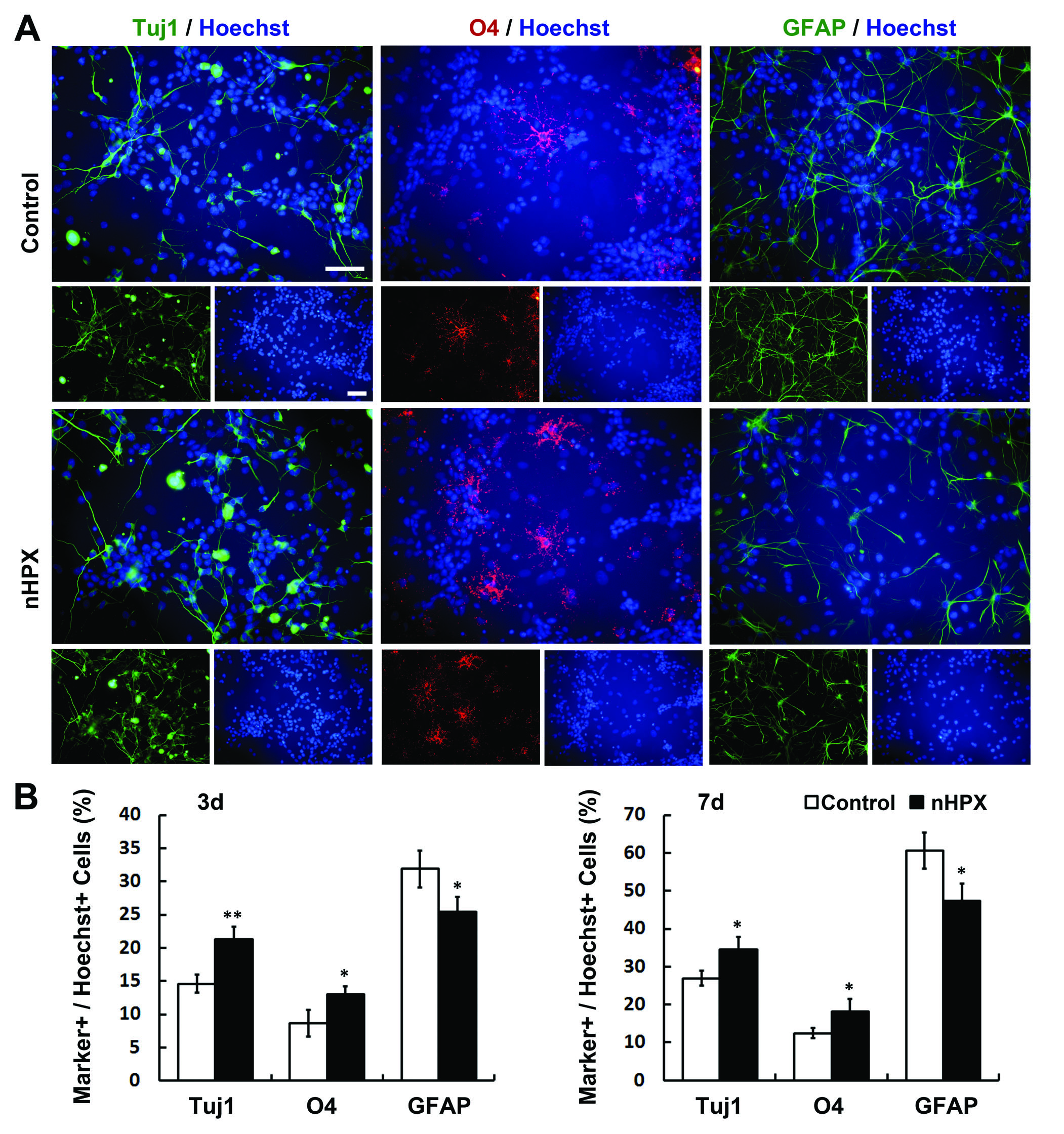
**

**Figure S3.** Hemopexin promotes cultured SVZa stem cells/ progenitors to differentiate into neurons and oligodendrocytes in vitro. (A) SVZa stem cells/ progenitors were cultured in DM or with nHPX for 3 d. Cells were labelled with Tuj1 (green), O4 (green) or GFAP (red). Hoechst (blue) was used to label nuclei. (B) The percentage of Tuj1+, O4+, and GFAP+ cells was calculated in each group after cells were cultured with nHPX for 3 d or 7 d, respectively. More Tuj1+ and O4+ cells but fewer GFAP+ cells were observed in the nHPX group. N=3. *p<0.05, **p<0.01. Scale bar: 50 µm.

**
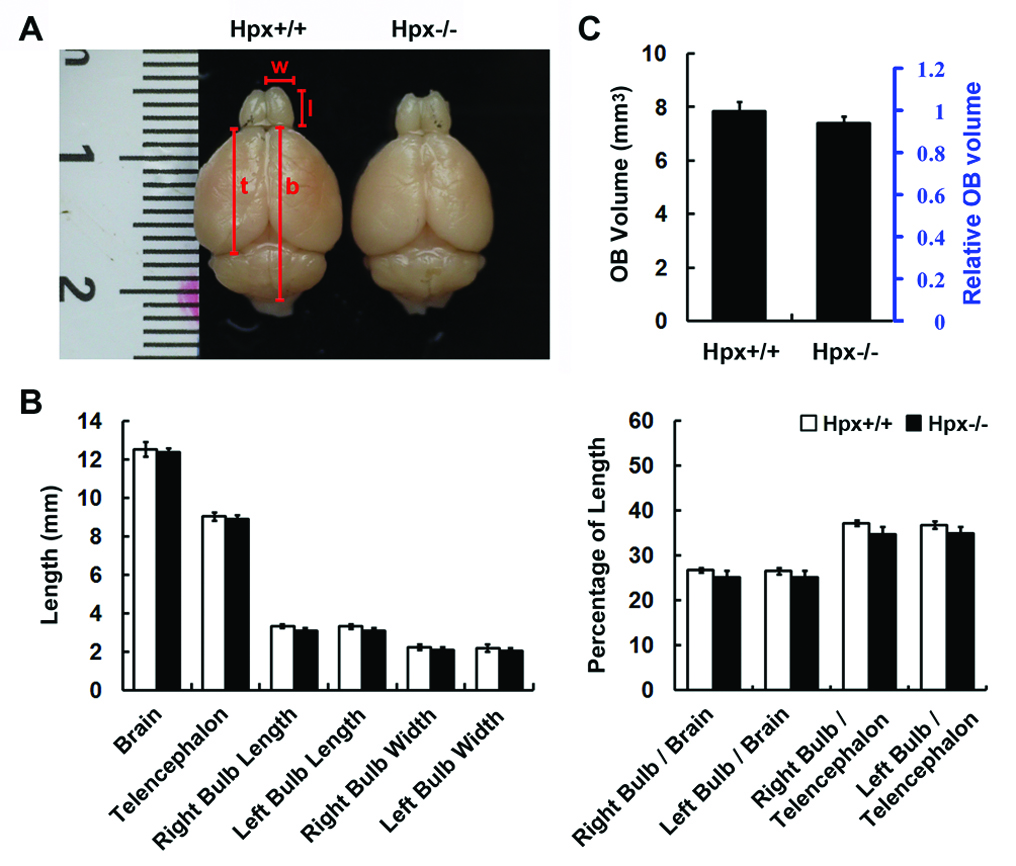
**

**Figure S4.** Hemopexin deletion did not affect the shape of the brain and the OB, neither did the volume. (A) Brains from 10-week-old Hpx+/+ (n=11) and Hpx-/- (n=9) mice were dissected and imaged. (B) Measurements were taken for brain, telencephalon, and olfactory bulb length and for olfactory bulb width. The percentage of bulb length compared to the length of the brain and telencephalon was calculated. No significant difference was found in the shape of brain, telencephalon or olfactory bulb between Hpx^+/+^ and Hpx^-/-^ mice. (C) Unbiased stereological measurements were performed to measure the size of the OB (n=5 per genotype). No significant difference was observed in the volume of the OB.

**
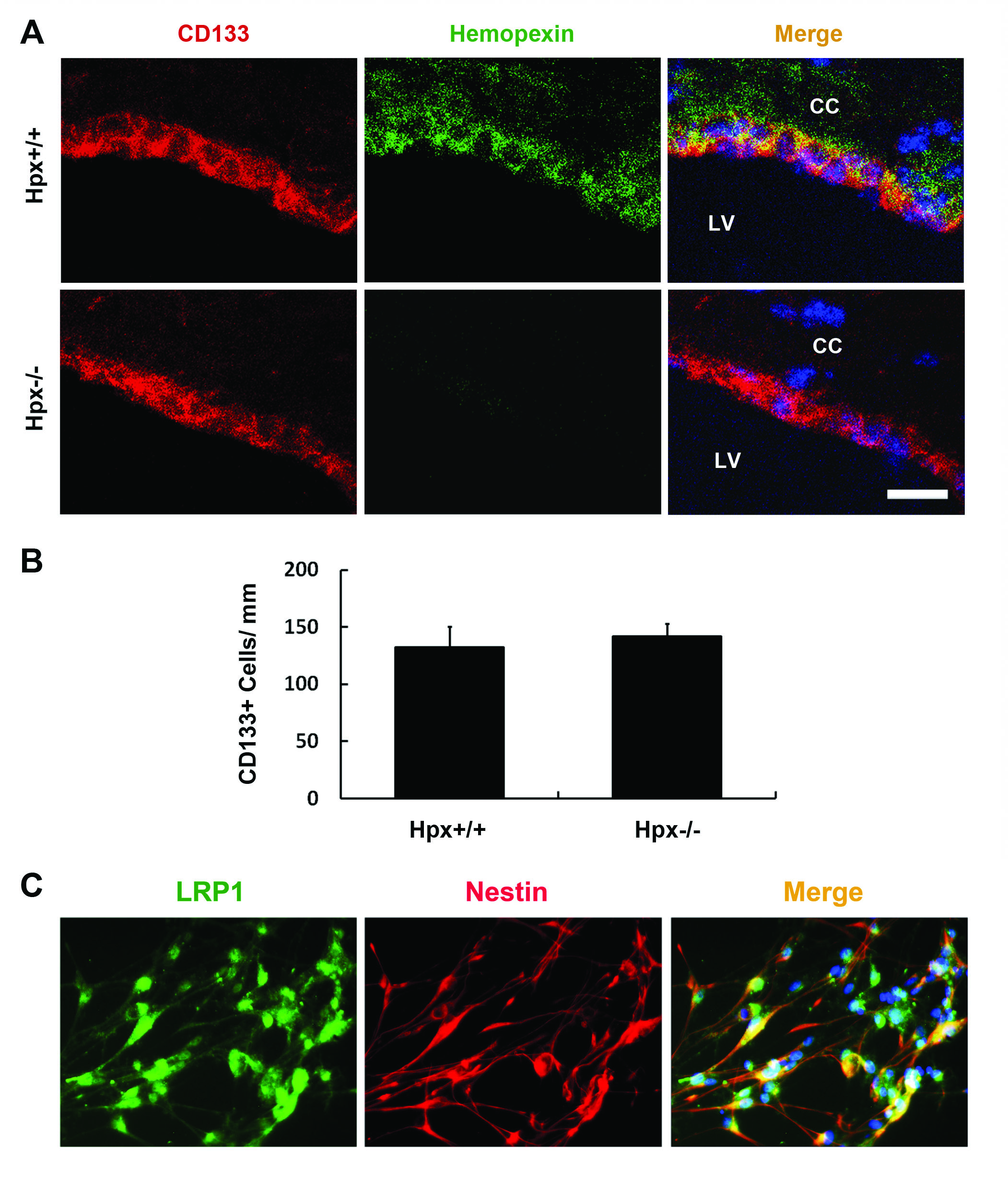
**

**Figure S5.** (A-B) Hemopexin expression in ependymal cells in wild type and Hpx-/- littermates. Coronal sections were prepared from 10-week-old brains. Hemopexin antibody was used to label hemopexin, and CD133 antibody was used to label ependymal cells. Hoechst staining (blue) was used to identify nuclei. CD133+ cells were co-labeled with anti-hemopexin in Hpx+/+ mice, while few CD133+ ependymal cells were hemopexin-positive in Hpx-/- mice (A). Number of CD133+ cells in the SVZ was counted (B). No significant difference was observed in the number of ependymal cells between Hpx+/+ and Hpx-/- mice. And the alinement of CD133+ ependymal cells in Hpx-/- mice was as normal as that in the wild type. LV, lateral ventricle; CC, corpus callosum. Scale bars: 25µm. (C) LRP1 was expressed in the cultured SVZ stem cells. SVZa neurospheres were dissociated, and single cells were cultured for 24h. Cells were labelled with LRP1 antibody (green), Nestin antibody (red) , and hoechst (blue) was used to label nuclei. As shown, most of the Nestin+ stem cells were also LRP1+.


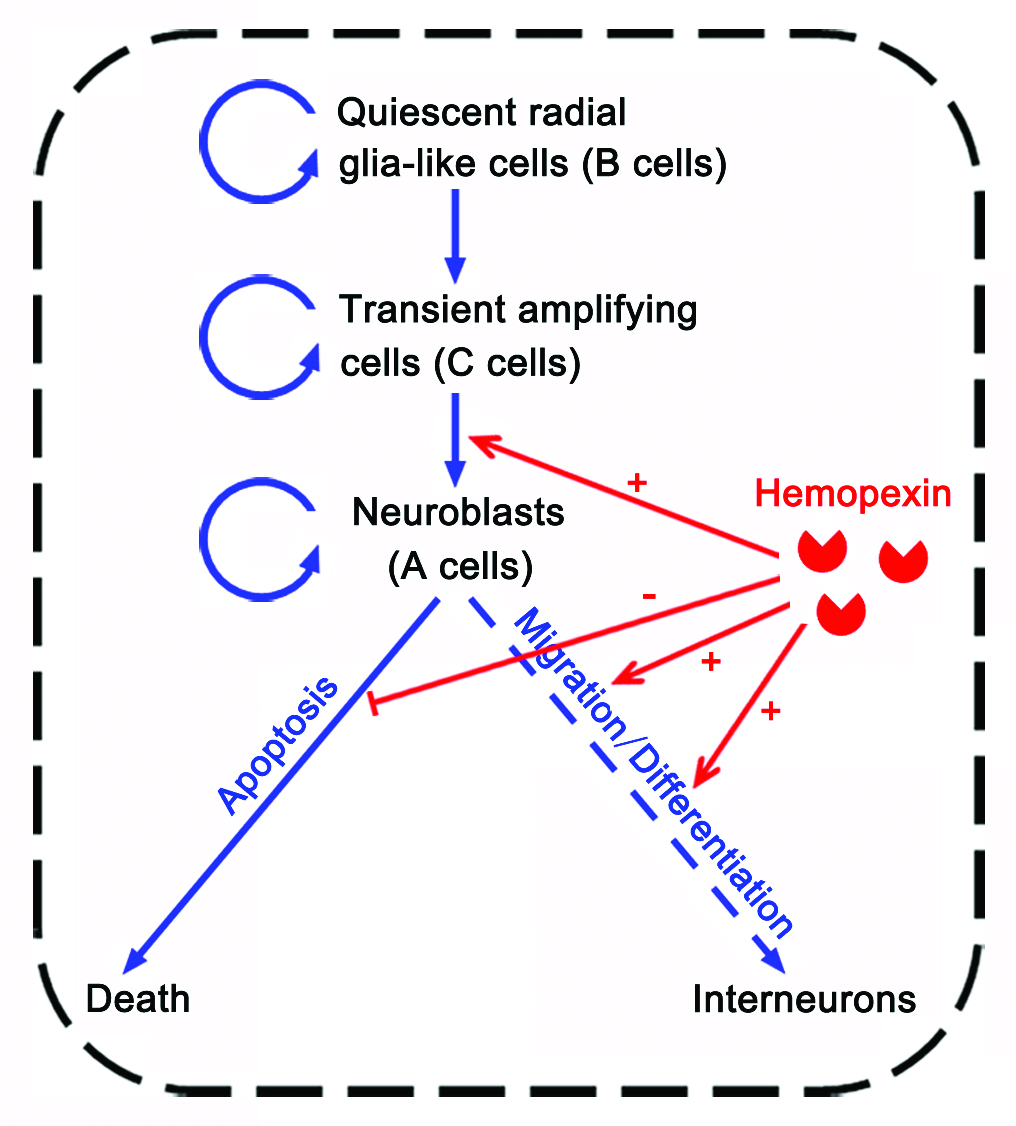


**Figure S6**. A model of lineage relationship of Neural Stem Cells (NSCs) and the effects of hemopexin in the Adult SVZ/OB pathway. NSCs / progenitors in the SVZ are classified as three major types: quiescent radial glia-like cells (B cells), transient amplifying cells (C cells) and neuroblasts (A cells). Type B cells slowly divide and produce Type C cells, while type C cells in turn give rise to Type A cells. The Type A cells migrate anteriorly in chains along the RMS to the OB, where they differentiate into interneurons (blue arrows). Hemopexin promotes the migration and differentiation of NSCs/progenitors, and inhibits the apoptosis of neuroblasts (red arrows).

**Supplementary Experimental Procedures**

**Size measurement of brain regions**

Brains from 10-week-old Hpx^+/+^ (n=11) and Hpx^-/-^ (n=9) mice were dissected and imaged. Measurements were taken for brain, telencephalon, and olfactory bulb length and for olfactory bulb width. The percentage of bulb length compared to the length of the brain and telencephalon was calculated.

Unbiased stereological measurements (Zou et al., 2012) were performed to measure the size of the OB (n=5 per genotype). Every fifth serial 14 µm-thick coronal section of the OB (70 µm intervals) was stained with cresyl violet and used for measurements. The area of each section was measured. The volume of the OB was evaluated with the equation ∑ (i=0 to n)Ai d, where A equals the area of the i^th^ section, d is the distance between sections, and n is the total number of sections measured.
